# Expanding the Diagnostic Use of PCR in Leptospirosis: Improved Method for DNA Extraction from Blood Cultures

**DOI:** 10.1371/journal.pone.0012095

**Published:** 2010-08-11

**Authors:** Steen Villumsen, Rebecca Pedersen, Karen Angeliki Krogfelt, Jørgen Skov Jensen

**Affiliations:** Department of Microbiological Surveillance and Research, Statens Serum Institut, Copenhagen, Denmark; Charité-Universitätsmedizin Berlin, Germany

## Abstract

**Background:**

Leptospirosis is a neglected zoonosis of ubiquitous distribution. Symptoms are often non-specific and may range from flu-like symptoms to multi-organ failure. Diagnosis can only be made by specific diagnostic tests like serology and PCR. In non-endemic countries, leptospirosis is often not suspected before antibiotic treatment has been initiated and consequently, relevant samples for diagnostic PCR are difficult to obtain. Blood cultures are obtained from most hospitalized patients before antibiotic therapy and incubated for at least five days, thus providing an important source of blood for PCR diagnosis. However, blood cultures contain inhibitors of PCR that are not readily removed by most DNA-extraction methods, primarily sodium polyanetholesulfonate (SPS).

**Methodology/Principal Findings:**

In this study, two improved DNA extraction methods for use with blood cultures are presented and found to be superior in recovering DNA of *Leptospira interrogans* when compared with three previously described methods. The improved methods were easy and robust in use with all tested brands of blood culture media. Applied to 96 blood cultures obtained from 36 patients suspected of leptospirosis, all seven patients with positive convalescence serology were found positive by PCR if at least one anaerobic and one aerobic blood culture, sampled before antibiotic therapy were tested.

**Conclusions/Significance:**

This study suggests that a specific and early diagnosis can be obtained in most cases of severe leptospirosis for up to five days after initiation of antimicrobial therapy, if PCR is applied to blood cultures already sampled as a routine procedure in most septic patients.

## Introduction

*Leptospira* species is the causative agent of the leptospirosis, one of the world's most wide-spread zoonosis [1]. Signs and symptoms of the disease are often non-specific and range from flu-like symptoms to multi-organ failure [1]. Carrier animals excrete the bacteria in large numbers with the urine and transmission to humans occurs mainly through contact with water or crops contaminated with infected urine [1]. The disease is endemic in developing countries mainly in the tropics where outbreaks occur frequently after heavy rainfalls [2]. Travellers may be exposed during activities in fresh water, and leptospirosis has recently been shown to be a relatively common cause of fever in Swedish travellers [3]. However, only few cases of leptospirosis are diagnosed every year in the developed countries. These cases are likely to represent an underestimate, since the diagnosis can only be established by leptospira specific tests. The gold-standard in the diagnosis of *Leptospira* spp. is detection of specific antibodies by the microscopic agglutination test (MAT) [1]. In most cases, a diagnostic serum sample can not be obtained before the 7^th^ day of disease and the diagnosis is thereby delayed for the same period. In the early phase of the disease, a rapid diagnosis can be obtained by PCR of *Leptospira* spp. This method has a sensitivity of 28–96% in severe leptospirosis when applied to whole blood samples [4], [5]. However, to ensure a high sensitivity, samples have to be obtained before or shortly after the start of antibiotic therapy, since antimicrobials quickly remove *Leptospira* spp. from the blood. In a non-endemic area, leptospirosis is rarely a first line diagnosis, and as symptoms can be severe, antibiotic treatment is often initiated before leptospirosis is suspected. It is, therefore, often impossible to obtain a relevant sample for diagnostic PCR.

In a hospital setting, blood cultures (BCs) are sampled from most septic patients before antimicrobial therapy is initiated and incubated for at least five days. BCs are closed container systems and consequently not prone to DNA-contamination but they contain inhibitors of the PCR that requires special procedures to remove [6]. In previous studies where PCR has been applied to BCs, only microorganisms that actually multiply in the BCs have been targeted. Even though, the sensitivity of the assays is highly dependent on the DNA-recovery, only little has been done to optimize these procedures. In whole blood samples, a high recovery is especially important, since the density of bacteria is very limited. Further, only a small fraction equivalent to 5–10 µl of the original sample is included in the final PCR assay.

The aim of this study was to evaluate five DNA extraction methods for their effectiveness in recovering *Leptospira* DNA and in removing inhibitors from spiked BCs. Moreover, we aimed to evaluate if BCs sampled before antimicrobial therapy could be used in the diagnosis of leptospirosis.

## Materials and Methods

### DNA extraction methods

The following five DNA extraction methods were used in the study:

#### Method 1 (M1)

DNA was extracted from 200 µl of the tested specimens using the DNeasy® Blood & Tissue Kit (Qiagen, Hilden, Germany) according to the manufacturer's instructions using the protocol for animal blood or cells. The final elution of DNA was done in 200 µl buffer AE.

#### Method 2 (M2)

DNA was extracted from 200 µl of the tested specimens using the MolYsis Plus Kit (Molzym GmbH & Co. KG, Bremen, Germany) according to the manufacturer's instructions for direct bacterial DNA isolation from blood culture. The final elution of DNA was done in 100 µl buffer EB.

#### Method 3 (M3)

DNA was extracted from 100 µl of the tested specimens using the method described by Fredricks and Relman [6]. Briefly, cell lysis was obtained by a guanidine hydrochloride based buffer. SPS (sodium polyanetholesulfonate) was removed by adding benzyl alcohol to the solution and subsequently separated from the aqueous phase containing the DNA by centrifugation. DNA was precipitated from the supernatant by sodium acetate in isopropanol. The pellet was “washed” in 70% ethanol and again resuspended in 100 µl Tris-EDTA buffer.

#### Method 4 (M4)

Benzyl alcohol based removal of SPS and other inhibitors in M3 was combined with the column based DNA extraction of M1 in a new protocol, M4. A 100 µl aliquot of the specimen was mixed with 100 µl lysis buffer (5 M UltraPure™ guanidine hydrochloride (Invitrogen, CA) in 100mM UltraPure™ Tris-HCl (pH 8.0; Invitrogen, Paisley, UK) and 10 µl proteinase K (20 mg/ml; Qiagen) and incubated for 10 minutes at room temperature. A total of 400 µl ultrapure water (Invitrogen) was added followed by 800 µl >99% benzyl alcohol (ReagentPlus®, Sigma-Aldrich, Brøndby, Denmark) and mixed. In order to separate the phases, the sample was then centrifuged at 20.000×g for 5 minutes at room temperature and 200 µl of supernatant (the aqueous phase) was transferred to a new tube as described in method 1, except that no proteinase K was added and that the sample was not incubated during the lysis step. Only 100 µl of buffer AE was added in the final elution step. This method was developed to be used with anaerobic and paediatric blood culture media.

#### Method 5 (M5)

This method was equal to M4 except that 600 µl of ultrapure water was added before the phase separation. This method was developed for use with all blood culture media.

### Experimental set-up

**Figure 1:**
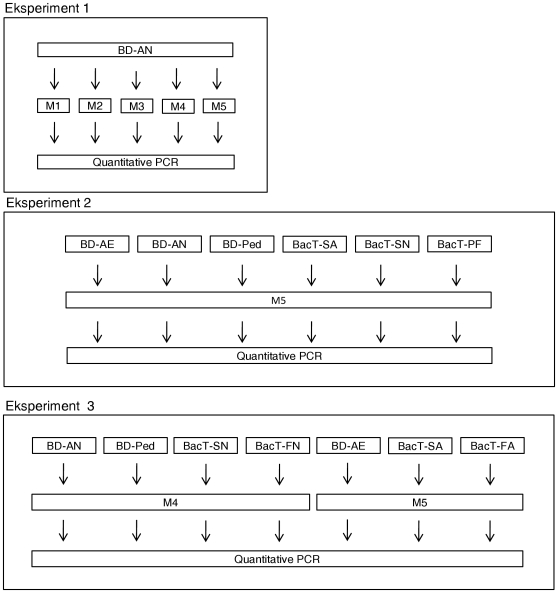
Flowchart for experiment 1–3. Figure shows flow over three independent experiments performed in the evaluation of five methods for DNA extraction from blood cultures media. BD-AE: BACTEC™ aerobic Plus, BD-AN: BACTEC™ anaerobic Plus, BD-Ped: BACTEC™ Paed Plus, BacT-SA: BacT/ALERT® SA, BacT-SN: BacT/ALERT® SN,BacT-PF: BacT/ALERT® PF, BacT-FA: BacT/ALERT® FA, BacT-FN: BacT/ALERT® FN. M1: Qiagen, DNeasy® Blood & Tissue Kit; M2: Molzym, MolYsis Plus Kit; M3: Protocol described by Fredricks and Relman; M4: Improved method for anaerobic and paediatric blood culture media; M5: Improved method adapted for all blood culture media.

The study consists of three experiments outlined in figure 1.

#### Experiment 1; evaluation of M1–M5 applied to one sample

One sample of BACTEC™ anaerobic Plus (BD diagnostics, NJ) collected from an anonymous patient for diagnostic purposes where no bacterial growth was detected after at least 5 days of incubation at 37°C was spiked with *Leptospira interrogans* (senso stricto) serovar Icterohaemorrhagiae strain M695 (available from KIT Biomedical Research, WHO/FAO Collaborating centre for Reference and Research on leptospirosis, the Netherlands). The bacterium was grown in Difco™ Leptospira medium - EMJH (Becton-Dickinson, Le Pont de Claix, France) until late log phase with a final concentration of 1.4×10^6^ *Leptospira*/µl. Bacterial cells were counted by dark field microscopy using a Helber counting chamber (Hawksley, Lansing, UK) as described by the manufacturer. The quantified culture was then frozen at −80°C in smaller aliquots. Just before use, one aliquot was thawed and added to blood culture material to yield a final concentration of 10,000 *Leptospira*/µl. The concentration of bacteria in the spiked samples was chosen to be at a level that would allow quantification of the recovery in methods with high and low recovery. The spiked sample was simultaneously extracted 10 times by each of the evaluated methods, M1–M5. The recovery of *Leptospira* DNA was assessed in triplicates by the quantitative PCR (qPCR) assay, described below. A total of 30 qPCR assays was made for each evaluated method. All DNA extractions were performed simultaneously.

#### Experiment 2; evaluation of M5 applied to six different blood cultures media

In pre-study tests of M4, problems of carry-over of inhibitors to the final eluate were observed when M4 was applied to BACTEC™ aerobic Plus and BacT/ALERT® SA (data not shown). The problem was identified as a very small water phase in the phase separation step leading to carry-over of the benzyl-alcohol phase on to the subsequent DNA extraction. The changes made in M5 completely solved all problems of carry-over of inhibitors and for that reason M5 is used in this experiment.

BACTEC™ anaerobic Plus, BACTEC™ Paed Plus, BACTEC™ aerobic Plus (BD), BacT/ALERT® SA, BacT/ALERT® SN and BacT/ALERT® PF (BioMerieux Inc., NC) collected for diagnostic purposes as described under experiment 1 were obtained from two Danish departments of clinical microbiology (Hillerød Hospital, Hillerød, Denmark and Statens Serum Institut, Copenhagen Denmark). For each of the six BC media tested, material from five different anonymous patients was obtained and spiked with *Leptospira* as described under experiment 1. DNA was extracted once by M5 from each of the 30 spiked samples. All DNA extractions were performed simultaneously. The recovery of *Leptospira* DNA was assessed as triplicates by qPCR, as described below.

#### Experiment 3; clinical evaluation of M4 and M5

Clinical material: Statens Serum Institut (SSI) is the only laboratory performing routine diagnostic testing for *Leptospira* in Denmark. In the period from January 2005 to June 2008, all BCs submitted for culture of *Leptospira* for diagnostic purposes were inoculated in Difco™ Leptospira medium - EMJH in order to isolate *Leptospira*, and the remainder of the BCs was stored at −20°C. When diagnostic follow-up had been completed, the patient identifications were removed from the blood cultures and the samples linked to the results of corresponding MAT and culture by a unique, anonymous code and included in the “Leptospira: PCR diagnostics” material collection (Danish Data Protection Agency: journal number 2010-41-4862).

Some of the BCs had been incubated for at least 5 days at 37°C at the local clinical microbiology laboratory before they were sent to SSI for isolation of *Leptospira* spp. In all of these cases, contact with SSI and the local clinical microbiology laboratory had been established for diagnostic reasons, and samples were only submitted if sampled before initiation of antimicrobial therapy. Ninety-six blood cultures (BACTEC™ aerobic Plus n = 20; BACTEC™ anaerobic Plus n = 21; BACTEC™ Paed Plus n = 2; BacT/ALERT® SA n = 23, BacT/ALERT® SN n = 21; BacT/ALERT® FA n = 8; BacT/ALERT® FN n = 1) were obtained from 36 patients (4 (11%) females and 32 (89%) males; median age 38 years; range 2 years to 78 years). Twenty-nine of these patients had a serum sample tested for antibodies against *Leptospira* spp. at some point during the acute phase of the disease. Ten patients had a repeated test for antibodies taken during convalescence.

Reference method: The test for antibodies against *Leptospira* spp. in a convalescence serum sample by a microscopic agglutination test (MAT) is a recognised reference standard in the diagnosis of leptospirosis [1]. Where requested by the clinicians, serum samples were tested for antibodies against *Leptospira* spp. by a ISO 17025 certified microscopic agglutination test (MAT) conducted as previously described by experienced laboratory technicians [7]. The MAT included *Leptospira* spp. of the following 13 serovars: Patoc, Icterohaemorrhagiae (3 strains), Sejroe (2 strains), Saxkoebing, Javanica, Canicola, Ballum, Bratislava, Grippotyphosa, Pomona, Bataviae, Hardjo and Hurstbridge. The following dilutions of sera (in titer): 30, 100, 300, 1,000, 3,000, 10,000, 30,000 and 100,000 were tested by MAT. When only a single serum sample was available for MAT, the test was considered positive if the highest observed titer was above 1,000 and possibly positive if the highest titer was with-in the range of 100–300. When two serum samples were available and these were obtained up to 4 weeks apart in the acute phase of the disease, then the test was considered as positive when a seroconversion from no reaction to a titer of at least 100 or a rise in the highest titer of two titer-steps, was observed. If no change or a change of only one titer-step were observed, the results were interpreted according to the results of the last sample as described above. Finally, the test was classified as false positive, if a decrease in titer of two titer steps or more were observed during this period, a finding sometimes observed with this test. All serological tests and culture were conducted before the results of the PCR assays were known.

Procedure for DNA extraction and qPCR: DNA extraction was performed by M4 for anaerobic and paediatric BCs while M5 was applied to the aerobic BCs. Recovery of *Leptospira* DNA was assessed as duplicates by qPCR as described below. M4 and M5 were evaluated for their ability to remove inhibitors of the qPCR by comparing the Ct value of the no-template controls (NTC) with the Ct value of samples where no amplification of the target DNA occurred.

### Ethical considerations

Exemption for review by the ethical committee system and for obtaining informed consent was obtained from the Committee on Biomedical Research Ethics for Capital Region (protocol number H-1-2010-FSP-20) in accordance with Danish law on quality development projects.

### Quantitative PCR assay

The *Leptospira* sp. DNA recovery of each extraction was assessed by a quantitative PCR (qPCR) assay with previously described primers and probe [8]. An in-house master mix was used [9] and included an internal amplification control (IAC) designed according to principles previously described [9]. All assays were performed on an Applied Biosystems 7500 quantitative PCR System platform using a previously described protocol [9]. Strict precautions to avoid PCR product carry-over was observed according to principles previously described [10]. A standard curve was constructed from 10-fold dilutions of purified genomic DNA of *L. interrogans* serovar Icterohaemorrhagiae strain M695 ranging in concentrations from approximately 100,000 leptospira DNA-copies to 1 leptospira DNA-copy/assay by principles previously described [9]. The limit of quantification (LOQ) of the assay was 100 *Leptospira* sp. DNA copies/assay and the limit of detection was <10 *Leptospira* sp. DNA copies/assay. Ultrapure water (Invitrogen) was used as NTC. Seven to eight NTC were included on each of the three qPCR plates used in the experiment. The qPCR targets *Leptospira* spp. specific sequences of the 16S rRNA gene present in two copies in the genome [1], [8]. The IAC included in the assay made it possible to detect even partial inhibition in assays, where no amplification of the target DNA occurred.

### Statistical analysis

The effect on the DNA-recovery by five different extraction methods (n = 50) and the six different blood culture systems (n = 30) was estimated by the copy-number and was assessed by multiple t-tests on log-transformed data (Log_n_+1) under Bonferroni multiple-comparison-test (LSMEANS analysis associated with PROC GLM, SAS 9.1; SAS Institute, US). The effect of blood culture media on the Ct value of the IAC was compared by a two-sample t-test of the mean for each PCR plate.

## Results

### 

#### Experiment 1

**Figure 2:**
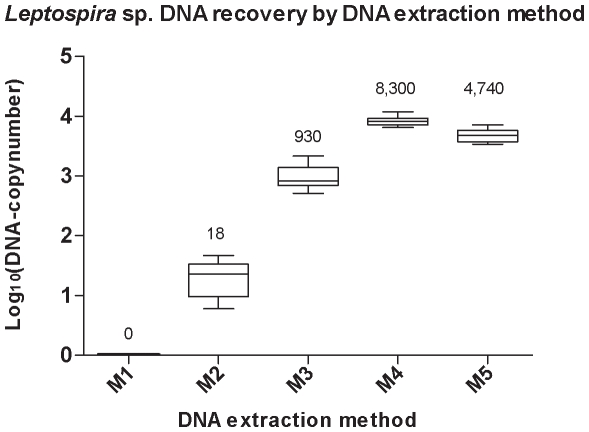
*Leptospira* sp. DNA recovery and inhibition of PCR. Estimated total copy-number of *Leptospira interrogans* DNA recovered from a spiked sample of BACTEC™ anaerobic Plus containing approximately 50.000 *Leptospira*/5 µl by five DNA extraction methods. The mean copy-numbers are given above each column. Error bars indicate the 95% confidence interval. M1: Qiagen, DNeasy® Blood & Tissue Kit; M2: Molzym, MolYsis Plus Kit; M3: Protocol described by Fredricks and Relman; M4: Improved protocol for anaerobic and paediatric blood culture media; M5: Improved protocol adapted for all blood culture media.

The results of experiment 1 are shown in figure 2 and table 1. M2–M5 removed inhibitors of the PCR to an extent that allowed amplification of *Leptospira* DNA in all of the corresponding qPCR assays. When M1 was applied to the samples, no amplification of *Leptospira* DNA was possible and the IAC was only amplified in two of the 30 corresponding qPCR-assays.

**Table 1:** Effectiveness of DNA extraction method, M1–M5 in recovering *Leptospira* DNA from BACTEC™ anaerobic Plus.

DNA-extration method	DNA-extractions	Positive quantitative PCR	Positive internal amplification control	*Leptospira* DNA recovered in percent of M4 (95% confidence interval)	P-value
**M1**	10	0	2	0% (0 to 0.02%)	P<0.001
**M2**	10	30	30	0.22% (0.16% to 0.29%)	P<0.001
**M3**	10	30	0	11% (9% to 14%)	P<0.001
**M4**	10	30	0	—	—
**M5**	10	30	0	58% (44% to 74%)	P<0.031

M1: Qiagen, DNeasy® Blood & Tissue Kit; M2: Molzym, MolYsis Plus Kit; M3: Protocol described by Fredricks and Relman; M4: Improved method for anaerobic and paediatric blood culture media; M5: Improved method adapted for all blood culture media.

The highest effectiveness in recovering *Leptospira* DNA was obtained by M4, while M5 resulted in a slightly lower recovery, 58% (95% CI, 44% to 74%; p<0.031) of M4. Due to the increased dilution of the sample in M5 compared to M4, a recovery of 76% was expected. Both methods were equally simple to use and the results were highly reproducible.

The three established methods (M1–M3) all had a significantly lower effectiveness in recovering *Leptospira* DNA than both M4 and M5. For M3, this was reduced to 11% (9%–14%; p<0.001) of M4 and for M2 only 0.22% (95% CI, 0.16% to 0.29%; p<0.001) of M4.

#### Experiment 2

**Figure 3:**
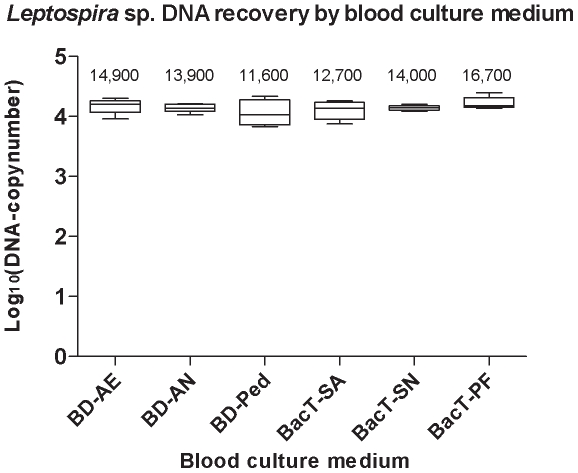
DNA recovery from different blood culture media. Estimated total copy-number of *Leptospira interrogans* DNA recovered from spiked samples of six different blood culture media containing approximately 50.000 *Leptospira*/5µl. All DNA extractions were performed with M5, the improved protocol adapted for all blood culture media. The mean copy-numbers are given above each column. Error bars indicate the 95% confidence interval. BD-AE: BACTEC™ aerobic Plus, BD-AN: BACTEC™ anaerobic Plus, BD-Ped: BACTEC™ Paed Plus, BacT-SA: BacT/ALERT® SA, BacT-SN: BacT/ALERT® SN and BacT-PF: BacT/ALERT® PF.

Results of experiment 2 are shown in figure 3. There was no sign of inhibition of the qPCR in any of the tested specimens, when M5 was applied to all of the tested blood culture media. There was no significant difference (n = 30; p = 0.54) in the *Leptospira DNA*-recovery obtained between the six different blood culture media tested.

#### Experiment 3

Seven of the 36 patients were diagnosed with leptospirosis by serological findings of either a single serum MAT titer ≥1,000 (n = 6) or by seroconversion to a MAT titer of 100 in several serovars in an early convalescence serum sample (n = 1). In one of the seven patients, EMJH-subculture of one of the BCs (BACTEC™ aerobic Plus) was found positive for *Leptospira borgpetersenii* senso stricto. From the remaining 29 patients, a serum sample was available from 21.In five of the 21 patients, a repeated serum sample taken and tested by MAT during convalescence and in one of the 21 patients the primary sample was taken 12 days after the blood cultures. Of the 21 patients, two had a MAT titer in the acute phase of the disease of 100 and 300 respectively, but on repeated testing during convalescence these were normalized. These patients were classified as false positive by MAT.

All of the seven patients diagnosed with leptospirosis by serology were positive by qPCR. One additional patient was positive by qPCR, but negative by MAT. He had only one serum sample taken at the same time as the blood cultures, and thus, it is likely that antibodies were not present at the time of sampling. All the remaining patients were negative by qPCR.

In 7 of the 8 positive patients the DNA-recovery was below LOQ i.e. they contained less than 100 DNA copies pr. asssay. A total of 17 BCs were positive by qPCR. The approximate median recovery in all positive samples was 11 *Leptospira* DNA-copies pr. assay (range 1 to 262). In four patients, all of the 2–5 analysed BCs were positive, while in four patients, where the DNA-recovery was close to LOD, only half of the 2 or 4 tested BCs were positive. In one of these last four patients, only one of the duplicate qPCR assays was positive in one BC.

The Ct value for the IAC was recorded for all 158 qPCR assays performed on the 79 blood cultures (nBC) where no amplification of leptospira DNA occurred in any of the two corresponding PCR assays. The 79 BCs included BACTEC™ aerobic Plus, n = 17; BACTEC™ anaerobic Plus, n = 16; BACTEC™ Paed Plus, n = 2; BacT/ALERT® SA, n = 19, BacT/ALERT® SN, n = 19; BacT/ALERT® FA, n = 5; BacT/ALERT® FN, n = 1). The results were compared to the Ct value recorded from the NTC and the results shown in table 2. A total of 3 plates were used for testing all clinical samples by qPCR.

**Table 2:** Comparison of the mean Ct value of the internal amplification control in no-template controls and blood cultures tested negative for *Leptospira* sp. DNA.

qPCR run	No-template control			Negative blood culture			P-value
	n	Ct	SD	n	Ct	SD	
**1**	7	33.8	0.4	52	33.5	0.3	p>0.05
**2**	8	33.7	0.4	34	33.5	0.2	p>0.05
**3**	7	34.1	0.3	72	34.0	0.2	p>0.05

## Discussion

In this study we present two improved DNA extraction methods designed for extraction of DNA from blood culture media, M4 and M5. The amount of *Leptospira* sp. DNA recovered by these methods were 9 and 5 times higher, respectively than what was obtained by M3, the best performing of the three previously described methods. This difference can largely be explained by the use of a column based DNA extraction in M4 and M5 in contrast to the DNA precipitation method used in M3. The sensitivity of the diagnostic set-up was remarkable when M4 and M5 were validated on samples from patients suspected of leptospirosis. At least one BC, from all of the seven patients that were tested positive by convalescence serology was also positive by qPCR in at least one of the duplicate qPCR assay wells. In addition, one patient was positive by qPCR but never had convalescence serology performed. These findings indicate that a very high sensitivity close to 100% can be obtained by this diagnostic set-up. The results are comparable to what have been found in a recent study where qPCR was compared to culture of *Leptospira* spp. [5], but remarkably higher than the 28–50% sensitivity that has previously been reported when PCR has been applied to whole blood samples in severe leptospirosis and compared with MAT [4], [11]. Four factors are likely to be important for this difference. In this study, at least two blood samples from each of the qPCR positive patients were tested. Only in 4 of the 8 positive patients, all BCs were positive by qPCR and this indicates that it is important to test at least two samples. Further, in one patient only one of duplicate qPCR assay wells was positive close to LOD. Even though such a result in a clinical situation needs further confirmation, the diagnosis might have been missed if the qPCR assay had not been tested in duplicate. Previous studies do not report having tested more than one sample from each patient nor do they report having performed more than one PCR assay for each sample [4], [11]. Furthermore, in this study BCs were tested and not serum or whole blood samples and this might affect the number of *Leptospira* spp. present in the samples. Five of the eight PCR positive patients were positive in more than one BC. The recovery of *Leptospira* DNA from the aerobic and the anaerobic BC were comparable in four of these patients but in one patient, the recovery of *Leptospira DNA* in BacT/ALERT® SA and BacT/ALERT® FA was >10 times higher than what was obtained from BacT/ALERT® SN, sampled at the same time. This suggests that some multiplication of the bacteria had occurred. The use of BCs and time of incubation might, therefore, at least in some cases play a role in the sensitivity of the assay, but we did not record the exact incubation period. Lastly, DNA precipitation technique has been used in previous studies for DNA-extraction and as we have shown in experiment 1, this is likely to result in a lower recovery of DNA compared with the column based method used in M4 and M5. The low DNA-copy-number present in the clinical samples stresses the importance of optimal microbial DNA recovery. It is therefore likely that neither M2 nor M1 can be used for diagnostic PCR in leptospirosis.

Fredricks and Relman were the first to effectively solve the problems of removing all inhibitors of the PCR from BCs. They indentified the anti-coagulant sodium polyanetholesulfonate (SPS) present in a anaerobic media of BacT/ALERT® as a potent inhibitor of PCR not readily removed by most extraction methods [6] and came up with a benzyl alcohol based extraction method, M3 to solve this problem [6]. However, M3 is, in our experience, not very robust in a clinical setting, especially when applied to aerobic BCs and for that reason the method has previously been rejected [12]. M4 and M5 have been developed on the basis of M3. By incorporating Proteinase K treatment in the lysis step, increasing the speed of centrifugation to 20.000×g in the phase separation step and for M5 by further adding extra water in the phase separation step all problems of carry-over of inhibitors to the final DNA preparation were completely solved. Throughout all the experiments, M4 and M5 were very robust and easy to use and as they are based on column-based DNA extraction, both methods are amenable to automation.

The IAC is a very sensitive way to detect inhibitors of the PCR, but in none of the experiments were there any signs of inhibition of the qPCR assay when M4 and M5 were used. A more exact estimate of this was given in experiment 3, by comparing for each qPCR plate the Ct value of the IAC of the NTC with the Ct value of the IAC in the BC samples where no amplification of *Leptospira* spp. DNA occurred. These 79 BCs included 5 BCs containing charcoal. No significant difference was observed in any of the assays and, thus, no sign of inhibition of the qPCR assays (table 2). Furthermore, no significant difference between in the recovery of *Leptospira* sp. DNA was observed when M5 in experiment 2 was applied to 6 different blood culture media from a total of 30 different patients.

To our knowledge, only one study by Gebert et *al*. has previously addressed the issue of recovering low amounts of bacterial DNA from blood cultures [13]. In that study, a near full recovery of *Staphylococcus aureus* DNA was obtained by M2, but when the method was applied to *Escherichia coli* the recovery was somewhat lower. In general, Gram-negative bacteria like *E. coli* and especially *Leptospira* spp. have a more fragile cell wall than Gram-positive bacteria. In M2, human cells are lysed and the DNA removed in the initial steps. Preliminary results suggest that the low recovery obtained by M2 in our study partly can be explained by a huge loss of bacterial DNA during these procedures.

If used with the described or slightly modified protocols, it is likely that BCs can be used in the diagnosis of other fastidious microorganisms like *Coxiella burnetii* [13] and dengue virus [14] in the future. It is also likely that the protocols will find use in severe infections like meningitis, where antibiotic treatment sometimes foregoes sampling of BCs and there by prevents the causative agent from growing in the media. Preliminary results suggest that M4 and M5 can be applied to *Neisseria meningitidis* and *Streptococcus pneumoniae* with a similarly high DNA recovery (data not shown).

The diagnosis of leptospirosis in non-endemic countries will often rely on convalescence serology since relevant samples for diagnostic PCR are difficult to obtain. In this study, two improved, easy and robust DNA extraction methods are presented. Both methods effectively removed all inhibitors of the PCR and they were found superior to three previously described methods in recovering *Leptospira* sp. DNA from blood cultures. This study, further, suggests that most cases of severe leptospirosis can be diagnosed by specific PCR if the described DNA extraction methods are applied to blood cultures routinely obtained from most septic patients and incubated for at least five days. Consequently, in the future, most cases of severe leptospirosis can be diagnosed by PCR in the early phase of the disease.
